# Insight into Influenza: A Virus Cap-Snatching

**DOI:** 10.3390/v10110641

**Published:** 2018-11-16

**Authors:** Corey De Vlugt, Dorota Sikora, Martin Pelchat

**Affiliations:** Department of Biochemistry, Microbiology and Immunology, Faculty of Medicine, University of Ottawa, Ottawa, ON K1H 8M5, Canada; cdevlugt@outlook.com (C.D.V.); dorotasikora@yahoo.com (D.S.)

**Keywords:** influenza A virus, cap-snatching, prime-and-realign, promoter-proximal pausing

## Abstract

The influenza A virus (IAV) genome consists of eight single-stranded RNA segments. Each segment is associated with a protein complex, with the 3′ and 5′ ends bound to the RNA-dependent RNA polymerase (RdRp) and the remainder associated with the viral nucleoprotein. During transcription of viral mRNA, this ribonucleoprotein complex steals short, 5′-capped transcripts produced by the cellular DNA dependent RNA polymerase II (RNAPII) and uses them to prime transcription of viral mRNA. Here, we review the current knowledge on the process of IAV cap-snatching and suggest a requirement for RNAPII promoter-proximal pausing for efficient IAV mRNA transcription.

## 1. The Influenza A Virus

The influenza A virus (IAV) is a highly pathogenic virus responsible for yearly epidemics and significant worldwide mortality. Seasonal influenza is responsible for thousands of deaths annually through respiratory complications and poses a large economic burden to health care systems worldwide. IAV is divided into subtypes based on the presence of one of the 18 hemagglutinin (HA) variants and one of the 11 neuraminidase (NA) variants, two viral membrane surface proteins. For instance, the H3N2 subtype contains the variant 3 and 2 of HA and NA respectively. Although aquatic birds can act as natural hosts for all subtypes, only a restricted number of subtypes can infect humans, such as the recurrent H1N1 and H3N2, or the highly pathogenic H5N1 subtypes. Despite the existence of vaccines, their effectiveness is limited by the antigenic variations of HA and NA [1]. While there are some antiviral drugs available for the treatment and prophylaxis of influenza, the emergence of drug-resistant strains is a concern [2]. A better understanding of the molecular interactions between the virus and its host is essential to identify new targets for antiviral therapy.

IAV is a negative-sense single-stranded RNA virus of the Orthomyxoviridae family. The IAV genome consists of eight segmented genes of varying lengths, each of which encodes at least one protein [3]. Each individual viral RNA (vRNA) segment is associated with a protein complex. The 3′ and 5′ conserved ends are associated with the RNA-dependent RNA polymerase (RdRp), whereas the remainder of the vRNA is coated with multiple copies of the nucleoprotein (NP) [4,5]. Upon infection of the host cell, these viral ribonucleoprotein (vRNP) complexes are transported into the nucleus where both transcription and replication of each vRNA is carried out by the viral RdRp. Although replication is a primer-independent process involving a complementary RNA intermediate, synthesis of the viral mRNA is dependent on short, capped primers derived from nascent host RNA polymerase II (RNAPII) transcripts [6]. During a process known as “cap-snatching”, the viral RdRp interacts with the C-terminal domain (CTD) of RNAPII and cleaves the newly synthesized transcript 10–13 nucleotides downstream of the 5′ m7G cap structure. This capped oligonucleotide primer is then used to initiate transcription from the vRNA template. This results in products that are genetic hybrids of host and virus mRNAs.

## 2. The Viral RNA-Dependent RNA Polymerase

The IAV RdRp is a heterotrimeric complex comprised of polymerase basic protein 1 (PB1), polymerase basic protein 2 (PB2), and polymerase acidic protein (PA). PB1 forms the core of the polymerase [7]. It contains conserved motifs typical of RNA-dependent RNA polymerases [8,9]. The PB2 and PA subunits are involved in the cap-snatching process. PB2 binds to the 5′ cap of nascent transcripts while PA cleaves host pre-mRNA 10–13 nucleotides downstream of the cap structure.

**Figure 1:**
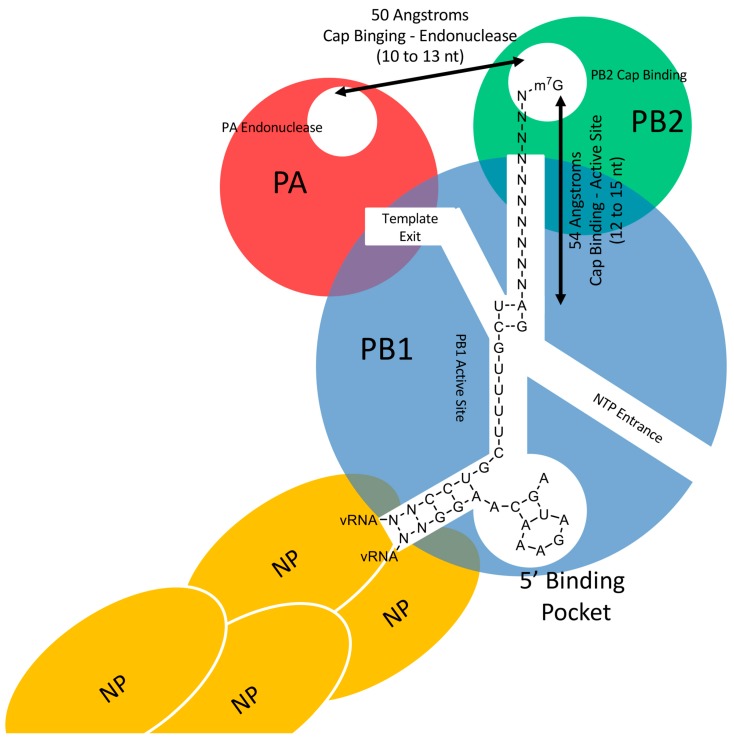
Cartoon representation of influenza A virus (IAV) RNA-dependent RNA polymerase (RdRp) bound to the viral RNA (vRNA) promoter and priming transcription. The PB1 subunit (blue) has two entrance tunnels, one occupied by the vRNA coming from the nucleoprotein (yellow), the other allowing nucleoside triphosphates (NTPs) to enter the active site. The two entrance tunnels join at the active site and split into the template exit tunnel (left, where the vRNA exits near PA opposite to the endonuclease) and the product exit tunnel. Cap snatched primers are threaded through the product exit tunnel to prime transcription. The distance from the PB2 cap binding region to the PB1 active site has been estimated to be 54 angstroms. The distance from the PB2 cap binding region to the endonuclease active site has been estimated to be 50 angstroms [11]. The 5′ end of the vRNA is tightly held in a binding pocket, and there are five base pairs between the 3′ end and the 5′ end of the vRNA. The nucleotides 3′-UCGUUUU are not bound to the polymerase. An A indicates an adenine residue, C indicates a cytidine residue, G indicates a guanine residue, U indicates a uridine residue, and N indicates a nucleotide residue. Nucleotides labeled as N within the vRNA are gene segment specific. Hashed lines indicate putative base pairs between the vRNA promoter and the host primer.

While the structure of the RdRp of a human-infecting strain of IAV remains to be determined, most of the structure of bat influenza A polymerase in complex with vRNA has been solved [7]. Inter-subunit interactions are extensive, with the three subunits being tightly interwoven. The buried area between PB1 and PA is estimated to be 17,330 angstroms^2^, between PB2 and PB1 14,100 angstroms^2^, and between PB2 and PA 2880 angstroms^2^ [7]. RdRp forms a “U” shape with PB1 forming the central portion and the cap-binding region of PB2 and the endonuclease-containing region of PA forming the upper portions [7] (see Figure 1 for a cartoon representation). This conformation is not static, however. The PB2 and PA subunits can undergo extensive conformational rearrangements when bound to the vRNA promoter, with the cap-binding and endonuclease domains aligned to allow cap-snatching [10]. 

## 3. Characteristics of the Host Primers Used during IAV Cap-Snatching

During the transcription pre-activation state, the viral RdRp cap-binding (on PB2) and endonuclease (on PA) domains are aligned in a way to promote cap-snatching [11]. Following cleavage, the capped primer is transferred to the vRNA template via the product exit tunnel. Complementarity between the capped RNA primer and the 3′ end of the vRNA template might stabilize the complex. Transcription initiates with the addition of a G or a C residue to the 3′ end of the capped primer, directed by the penultimate C residue (C2) or the G residue at position 3 (G3) in the vRNA template. Interestingly, some cap-snatched primers are not directly used to transcribe mRNA but instead undergo a prime-and-realign mechanism (PAR). PAR is a mechanism in which the capped RNA leader is first elongated by one to several nucleotides. This is followed by backward realignment of the nascent chain. This model explains the presence of extra repetitive sequences found at the end of the host-derived leaders in viral mRNA. About 14% of host-derived primers had repetitive sequences at the 3′ end that were likely generated by PAR [12].

The length of cap-snatched primers appears to be influenced by the distance between the cap-binding region (PB2) and the endonuclease region (PA) of the RdRp complex (Figure 1). This distance is 50 angstroms, or approximately 10 to 13 nucleotides [11], and is reflected in both the length of cleavage preference (10 to 13 nucleotides) [13], and the length of the population of cap-snatched primers in vivo (primarily between 10 and 13 nucleotides) [12]. While length is important in determining the range of the cleavage site [13], cleavage is sequence dependent within this length range. Selective cleavage of a sequence compatible with one or more nucleotides of the vRNA conserved sequence has been observed during cap-snatching [13,14,15,16,17]. Selective cleavage of host mRNA yields a nucleotide sequence that is generally complementary to nucleotides near the 3′ end of the vRNA. The IAV RdRp preferentially cleaves 3′ of a guanine residue within a 5′-GC-3′ motif [13] and directs this guanine to the 3′C2 of the vRNA template to initiate transcription internally rather than terminally [17,18]. Cleavage at a nucleotide sequence that allows for internal initiation appears to be the preference of the endonuclease rather than a sequence which allows for terminal initiation [12,14,15,16]. Furthermore, a nucleotide complementary to the first nucleotide of the vRNA conserved sequence (a uridine) is often absent in the mRNA, with transcription being primed at 3′C2 [14,15,16,17]. The internal initiation strategy might be selected as this has a lower free energy and faster rate of reaction than terminal initiation due to the overhanging nucleotide sequence [19].

Guanine and adenine are favored at the ultimate and the penultimate positions of the host-derived primer respectively (Figure 1) [6,13,14,15,16,20,21]. Selection of both the ultimate and penultimate residues is consistent with formation of base pairs between host primer and vRNA. Interestingly, priming of transcription without a complementary nucleotide has also been observed for IAV [13,16,17]. Primers lacking a guanine residue complimentary to the 3′C2 and/or a nucleotide complementary to the uridine residue at position 3′U1 have been observed to prime transcription [12,13,14,15,16,17,22,23]. This flexibility in priming nucleotide sequence seems to be conferred by the 3′U1, which was observed to form non-Watson–Crick base pairs with a U or G residue [17].

## 4. Cap-Snatching Is Based on Host mRNAs Abundance

When mRNA primers are provided in near equimolar amounts or in excess, cap-snatching occurs solely on the basis of sequence compatibility with the 3′ end of the vRNA, within the compatible length range [13,14,15]. These analyses were performed either in vitro or by examining only a small and specific population of mRNA that could serve as primers and do not likely represent the nature of this process in vivo. In vivo, there is a much larger pool of host transcripts to serve as cap donors and use of a transcript is subtractive. When mRNA is used to prime transcription, it is removed from the pool of potential cap donors. Despite limited studies examining cap-snatching on the basis of cap donor availability, this process appears to be related to the abundance of the RNA [12,16].

An initial analysis of cap-snatched mRNAs for A/HongKong/1/1968 (H3N2) indicated that this process is related to abundance as there is no significant difference of enriched gene ontology terms between cap-snatched mRNA and host cell mRNA levels [16]. However, non-coding RNAs were not analyzed in this study. Two subsequent studies using different IAV strains (A/WSN/33 (H1N1) [23] and A/Brisbane/59/2007 (H1N1) [22]) suggested that sequences corresponding to the very abundant small RNAs, such as snRNAs and snoRNAs, were cap-snatched preferentially and that correlation exists between host RNA abundance and cap-snatching by the A/Brisbane/59/2007 (H1N1) RdRp [22]. Reanalysis of these three data sets as well as an additional data set (A/PuertoRico/8/1934 (H1N1)) confirmed that the enrichment of most transcripts used as cap donors can be explained by the abundance of these RNAs, including the frequent use of snRNAs and snoRNAs [12].

There are, however, some RNAs that are enriched within the cap-snatched population for IAV beyond levels which can be directly explained by abundance, particularly protein coding genes related to cellular metabolism, protein localization, regulation of cell cycle, and apoptosis [12]. One potential explanation for this observation is the change in the cellular transcriptome in response to viral infection and host shut-off. IAV upregulates genes involved in metabolism and apoptosis in infected cells [24,25,26]. It is likely that the increased transcription of these mRNAs is directly related to their increased use as cap donors. The other possible explanation of the selective enrichment is that the enriched RNAs have a compatible nucleotide sequence within the preferred length range for IAV cleavage. Using transfected cells with plasmids containing a known sequence and varying degrees of complementarity to the vRNA template, it was previously shown that relatively less abundant mRNAs with a higher degree of sequence complementarity can be cap-snatched more frequently than a less compatible, relatively more abundant mRNA [14]. An enrichment of cap donors that could be cleaved within a 5′-AGC-3′ motif was observed in the A/Hong Kong/1/1968 (H3N2) data set [16] showing that this preference is likely maintained in the context of host derived transcripts and is consistent with the higher PA endonuclease activity 3′ of a guanine residue within a 5′-GC-3′ motif [13]. Taken together, these findings indicate that there may be a bias towards compatible primers within the abundant primer subset that lead to either an enrichment or a reduction in the levels of an RNA species used as a cap donor relative to host expression levels due to events occurring after RNAPII association or cap-binding.

## 5. IAV RdRp Associates with Host DNA-Dependent RNAPII during Cap-Snatching

Although the IAV RdRp can initiate transcription of mRNA from a free capped primer, in vivo cap-snatching involves association with RNAPII [6]. The first indication of RNAPII involvement in this process was the observation that treatment of cells with the RNAPII inhibitor α-amanitin also inhibited RdRp mediated transcription [27]. IAV RdRp was also shown to co-immunoprecipitate with RNAPII promoter DNA [28]. Subsequent studies demonstrated the association between RdRp and the CTD of serine-5-phosphorylated RNAPII, which corresponds to the initiating form of RNAPII [28,29,30,31,32]. Specifically, IAV RdRp interacts with the serine-5-phorphorylated form of RNAPII, but not the non-phosphorylated or the serine-2-phosphorylated, which is associated with elongating forms of RNAPII [28,31]. Additionally, a co-crystal structure of bat influenza A polymerase bound to an RNAPII CTD peptide mimic showed two distinct phosphoserine-5 binding sites in the PA subunit binding to four CTD heptad repeats [32].

The CTD of RNAPII is phosphorylated at serine-5 during transcription initiation. Soon after initiation, RNAPII pauses mRNA synthesis at a location 20–60 nucleotides downstream of the transcription start site in a large proportion of genes [33,34]. This process is called promoter-proximal pausing. During this pause, the DRB Sensitivity Inducing Factor (DSIF) and the negative elongation factor (NELF) bind actively engaged RNAPII and repress its elongation. The paused RNAPII remains stably associated with both the nascent pre-mRNA and DNA template and resumes elongation when the repression is reversed by P-TEFb. P-TEFb is a cyclin-dependent kinase that phosphorylates the repressive DSIF/NELF complex causing NELF to dissociate from the transcription complex and transforming DSIF into a state that stimulates RNAPII elongation. P-TEFb also phosphorylates serine-2 within the heptad repeat of the CTD. This creates a platform for binding RNA-processing factors and facilitates productive RNA synthesis. Promoter-proximal pausing coordinates mRNA capping with elongation, and correctly capped nascent mRNA is a prerequisite for escape from the pause [34,35]. By binding to RNAPII on genes during promoter-proximal pausing, IAV RdRp could easily gain access to the 5′ cap structure of the nascent transcripts. To support this hypothesis, it was found that immunodepleting the positive transcription elongation complex P-TEFb abolished the association of IAV vRNP with RNAPII and its cellular level correlated with viral transcription efficiency [36]. Also consistent with a role of promoter-proximal pausing is the observation that IAV efficiently cap-snatches small RNAs [12,22,23].

## 6. Conclusions and Challenges

The cap-snatching process seems to be governed by the structure of the vRNA-bound RdRp. Upon binding to the CTD of RNAPII, the cap-binding domain (PB2) binds to the 5′ cap of cellular RNAs. Cleavage occurs 10 to 13 nucleotides downstream of the 5′ cap structure, a length corresponding to the 50 angstrom distance between the cap-binding and endonuclease domains. The endonuclease cleaves primarily 3′ of a guanine residue within a 5′-GC-3′ motif. This capped oligonucleotide is used to prime transcription of viral mRNA either at 3′U1 or when the cap-snatched primer ends in a guanine residue at 3′C2. Primers are cap-snatched on the basis of abundance in vivo. Deviations from a direct correlation with abundance arise due to changes in the host transcriptome upon viral infection, or due to high sequence complementarity with the vRNA template. Essential to this process is the association with the CTD of serine-5-phosphorylated RNAPII, which corresponds to the initiating form of RNAPII. Either a reduction in RNAPII pausing by NELF depletion or a decrease in affinity of RdRp for RNAPII reduces cap-dependent viral transcription.

Many questions remain unanswered, such as how the RdRp is recruited to the paused (initiating) RNAPII. One study implicated P-TEFb as the factor involved in bringing the RdRp to the stalled transcription complex [36], but the interaction between RdRp and P-TEFb has not been corroborated by additional studies. Furthermore, despite recent advances in the structural characterization of the IAV RdRp, no comprehensive structure of the human IAV polymerase has been crystallized, and there is no crystal structure of the polymerase at initiation of transcription with a cap-snatched primer. Another challenge is determining the gene of origin for a given primer. This is difficult both due to the short length and the incomplete nature of annotated genomic transcription start sites. The determinants of the prime-and-realign process, which occurs for approximately 14% of primers, are also unknown.
